# Rivers as carriers and potential sentinels for *Burkholderia pseudomallei* in Laos

**DOI:** 10.1038/s41598-018-26684-y

**Published:** 2018-06-06

**Authors:** Rosalie E. Zimmermann, Olivier Ribolzi, Alain Pierret, Sayaphet Rattanavong, Matthew T. Robinson, Paul N. Newton, Viengmon Davong, Yves Auda, Jakob Zopfi, David A. B. Dance

**Affiliations:** 1 Lao-Oxford-Mahosot Hospital-Wellcome Trust Research Unit, Microbiology Laboratory, Mahosot Hospital, Vientiane, Laos; 2 Department of Environmental Sciences, University of Basel, Basel, Switzerland; 3 Swiss Tropical and Public Health Institute, University of Basel, Basel, Switzerland; 4 GET, Université de Toulouse, IRD, CNRS, UPS, Toulouse, France; 5 iEES-Paris (IRD, Sorbonne Universités, UPMC Univ Paris 06, CNRS, INRA, UPEC, 10 Université Paris Diderot), c/o Department of Agricultural Land Management (DALaM), Vientiane, Laos; 6 Centre for Tropical Medicine and Global Health, Nuffield Department of Medicine, University of Oxford, Oxford, UK; 7 Faculty of Infectious and Tropical Diseases, London School of Hygiene and Tropical Medicine, London, UK

## Abstract

*Burkholderia pseudomallei*, causative agent of the often fatal disease melioidosis, dwells in tropical soils and has been found in freshwater bodies. To investigate whether rivers are potential habitats or carriers for *B*. *pseudomallei* and to assess its geographical distribution in Laos, we studied 23 rivers including the Mekong, applying culture-based detection methods and PCR to water filters and streambed sediments. *B*. *pseudomallei* was present in 9% of the rivers in the dry season and in 57% in the rainy season. We found the pathogen exclusively in Southern and Central Laos, and mainly in turbid river water, while sediments were positive in 35% of the *B*. *pseudomallei*-positive sites. Our results provide evidence for a heterogeneous temporal and spatial distribution of *B*. *pseudomallei* in rivers in Laos with a clear north-south contrast. The seasonal dynamics and predominant occurrence of *B*. *pseudomallei* in particle-rich water suggest that this pathogen is washed out with eroded soil during periods of heavy rainfall and transported by rivers, while river sediments do not seem to be permanent habitats for *B*. *pseudomallei*. Rivers may thus be useful to assess the distribution and aquatic dispersal of *B*. *pseudomallei* and other environmental pathogens in their catchment area and beyond.

## Introduction

Knowledge of the distribution and dispersal of pathogens in natural environments is crucial to understand the epidemiology of the diseases they cause, improve risk models and develop effective health management strategies^1,2^, particularly in countries with limited economic resources. Dispersal of microbes, including pathogenic species, is facilitated by transport in water and air, on particles or passive carriers (e.g. migrating birds) or in vectors and hosts^3^. While most research on the fate and transport of water-borne pathogens focuses on enteric bacteria^4^, studies addressing dispersal mechanisms of pathogens with environmental reservoirs, for example *Burkholderia pseudomallei*, are rare. The soil-dwelling bacterium *B*. *pseudomallei* is an emerging human pathogen and causative agent of melioidosis, an underdiagnosed infectious disease with an estimated global incidence of 165,000 cases per year of whom approximately 50% die^5^. Mainly known in Southeast Asia and Northern Australia, a recent environmental suitability model predicted a widespread occurrence of *B*. *pseudomallei* in tropical soils throughout the world. Consequently, melioidosis is probably endemic in many countries where it has never been reported^6^. In soil, *B*. *pseudomallei* is spatially heterogeneously distributed across different scales, ranging from geographical regions to localised patches of a rice field^7^, which makes its detection challenging. In addition to soil, *B*. *pseudomallei* has been found in a range of freshwater sources, including drinking water in Thailand^8^ and Australia^9–11^ and a river in Lao People’s Democratic Republic (Laos)^12,13^, where the distribution of melioidosis remains uncertain. *B*. *pseudomallei* in freshwater bodies are potential sources of infection^9^, particularly if they live permanently in these habitats. Moreover, rivers may transport *B*. *pseudomallei* from sources in the watershed and thereby indicate the presence of *B*. *pseudomallei* in the catchment and act as carriers for its environmental dispersal.

The aims of this pilot study were to investigate (i) the geographical distribution of *B*. *pseudomallei* in Laos and (ii) whether rivers are potential reservoirs and/or carriers for *B*. *pseudomallei*. For this purpose, we used two independent methods, conventional culture and PCR after enrichment, to detect *B*. *pseudomallei* in river water and, for the first time, in streambed sediments, and assessed the distribution data in an environmental context to explain spatiotemporal variations.

## Results

**Figure 1:**
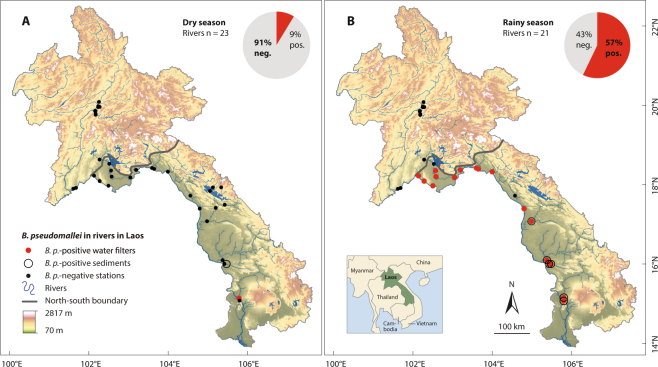
*B*. *pseudomallei* (*B*.*p*.)-positive and -negative stations and rivers in the dry season (**A**) and rainy season. (**B**) North-south boundary based on^38,39^, map background based on elevation data (U.S. Geological Survey, https://earthexplorer.usgs.gov; Central Intelligence Agency, https://www.cia.gov/library/publications/the-world-factbook/index.html) and rivers/lakes/country shapefiles provided by the Centre for Development and Environment (CDE), CDE Lao Country Office, Laos. Geographic coordination system: WGS 1984, latitude and longitude in degrees; altitude of highest and lowest point in meters above mean sea level.

We investigated 23 rivers (36 sampling sites, hereafter stations) in Laos between 15 °N and 20 °N, including the Mekong (Table 1). *B*. *pseudomallei* was present in 9% (2/23) of the rivers (2/36 stations) in the dry season. In contrast, we found the pathogen in 57% (12/21) of the rivers (17/31 stations) in the rainy season, detected on at least one water filter (pre- or main filter) by at least one detection method (conventional culture or PCR after enrichment; Table 1). Apart from one filter-negative, sediment-positive station in the dry season, we only found *B*. *pseudomallei* in the sediment when it was present in the water, i.e. in 35% (6/17) of the *B*. *pseudomallei*-positive stations in the rainy season. All *B*. *pseudomallei-*positive stations were situated in the centre and south of Laos, and *B*. *pseudomallei*-positive sediments were only detectable in the southern-most rivers (Fig. 1). The north-south trend was also observable in *B*. *pseudomallei*-positive rivers with sampling sites in both regions, i.e. the Mekong (six sites) and Nam Ngum (four sites), where the northernmost 1–2 stations were negative and the 3–4 southernmost stations positive. The seasonal and regional contrast regarding the presence of *B*. *pseudomallei* was statistically significant when comparing all stations or all rivers, as well as stations or rivers in the rainy season, and stations or rivers in the south (Fisher’s exact test, p ≤ 0.001).

**Table 1:** Sampled rivers and stations in Laos.

River	Tributary of	Stations	Region	Geographical coordinates		*B*. *pseudomallei*	
				Latitude	Longitude	D	R
Mekong	S. China Sea	6	N	19.95601	102.24113	−	−
			N*	17.89870	101.62397	−	−
			S*	17.97276	102.50410	−	+
			S*	17.39714	104.79999	−	+
			S*	16.00503	105.42449	−	+
			S	15.10721	105.79878	−	+
Nam Ou	Mekong	1	N	20.08642	102.26406	−	−
Nam Suang	Nam Pa	1	N	19.97931	102.24728	−	−
Nam Pa	Mekong	1	N	19.96049	102.28289	−	−
Nam Khan	Mekong	1	N	19.78600	102.18311	−	−
Houay Khan	Nam Khan	1	N	19.75995	102.18103	−	−
Houay Pano	Nam Khan	3	N	19.86034	102.17262	−	−
			N	19.85903	102.17061	−	−
			N	19.85263	102.16901	−	−
Nam Lik	Nam Ngum	1	N*	18.63280	102.28104	−	−
Nam Mi	Mekong	1	N*	17.91917	101.68856	−	−
Nam Ngum	Mekong	4	S*	18.52502	102.52631	−	−
			S*	18.35581	102.57204	−	+
			S*	18.20269	102.58588	−	+
			S*	18.17879	103.05593	−	+
Nam Thon	Mekong	1	S*	18.09152	102.28159	−	+
Nam Sang	Mekong	1	S*	18.22284	102.14222	−	+
Nam Mang	Mekong	1	S*	18.37019	103.19846	−	+
Nam Gniep	Mekong	1	S*	18.41756	103.60217	−	+
Nam Xan	Mekong	1	S*	18.39523	103.65408	−	+
Nam Kading	Mekong	1	S*	18.32517	103.99924	−	+
Nam Hinboun	Mekong	1	S*	17.72699	104.56798	−	−
Nam Xot	Nam Theun	1(0)	S*	17.93148	105.13257	−	nd
Nam Theun	Mekong or Xe Bangfai^†^	1(0)	S*	17.84229	105.05841	−	nd
Xe Bangfai	Mekong	3(1)	S*	17.49436	105.42959	−	nd
			S*	17.41563	105.20320	−	nd
			S*	17.07782	104.98496	−	+
Xe Banghieng	Mekong	1	S*	16.09804	105.37625	−	+
Xe Bangnouan	Mekong	1	S	16.00290	105.47937	+	+
Xe Don	Mekong	1	S	15.12390	105.80748	+	+

Stations: number of sampled stations in the dry season (rainy season in brackets if different). Region: geographical classification based on^38,39^; stations marked * belong to the centre of Laos (reference: Department of Tourism Marketing, Ministry of Information, Laos). *B*. *pseudomallei*: presence of *B*. *pseudomallei* by at least one detection method in river water and/or sediment. N = north, S = south, D = dry season, R = rainy season, nd = no data. ^†^Flow direction depends on water level regulations of the Nam Theun dam lake. Geographical coordinates in degrees (WGS 1984) (dry season).

Almost as many *B*. *pseudomallei*-positive stations were identified by conventional culture as by molecular techniques (Table 2). However, PCR revealed a higher number of positive samples per station than culture, and the only two *B*. *pseudomallei*-positive stations in the dry season were detected by PCR. All culture-positive sediments resulted from direct incubation of the highest volume of sediment fluid (500 µL) on Ashdown’s agar. *B*. *pseudomallei*-positive main filters (23/38) outnumbered pre-filters (15/38).

**Table 2:** Number of *B*. *pseudomallei* positive units comparing different detection methods and sample types (pre-filters, main filters, sediment).

*B*. *pseudomallei* positive units	Direct culture	Post-enrichment PCR	Both methods positive	Total
Stations (only by respective method)	15 (3)	16 (4)	12	19
All samples	16	31	10	47
Pre-filters	3	12	1	15
Main filters	10	13	7	23
Stations with positive pre- and main filter	0	10	0	10
Sediment samples	3	6	2	9
Filter-positive, sediment-negative stations	11	10	7	11
Sediment-positive, filter-negative stations	2	1	0	3
Stations where all samples were positive	0	4	0	4

The characteristics of physico-chemical water parameters measured on-site (turbidity, temperature, acidity, electrical conductivity as a proxy for salinity, dissolved oxygen, redox potential, altitude of the station) are shown in Table 3. Water temperature correlated moderately, and salinity, altitude, turbidity and pH weakly with the presence of *B*. *pseudomallei* on water filters (undirectional correlation). However, all physico-chemical parameters were functions of season and/or of region and correlated with at least one other parameter (Table 3). For example, water temperature was higher in the rainy season and in the south, and correlated negatively with altitude, while salinity showed the opposite pattern. As a result, none of the parameters was a significant independent predictor of the presence of *B*. *pseudomallei* in multivariate logistic regression models restricted to conditions under which *B*. *pseudomallei* was most common (in the water of southern river stations in the rainy season).

**Table 3:** Characteristics of physico-chemical water parameters and altitude.

Physico-chemical parameters	Median	Min – Max	Seasonal differences, mean (SD)		Regional differences in rainy season, mean (SD)		Corr. ratio	Bravais-Pearson correlations					
			Dry season	Rainy season	North	South	*B*. *p*.	Tur	Temp	pH	EC	DO	ORP
Altitude (m.a.s.l.)	175	74–513			322 (106)	140 (38)*	0.15*	0.01	−0.39*	0.02	0.52*	−0.47*	0.33
Turbidity (NTU)	18	2–730	12 (11)	227 (218)*	257 (242)	206 (206)	0.13*		0.35*	−0.21	−0.12	−0.07	−0.10
Temperature (°C)	26.5	19.5–30.0	25 (1.9)	27.6 (1.5)*	26.8 (1.8)	28.2 (1.1)*	0.29*			−0.17	−0.10	−0.04	−0.23
Acidity (pH)	7.6	6.5–9.0	7.8 (0.5)	7.3 (0.4)*	7.4 (0.4)	7.3 (0.4)	0.09*				0.40*	0.34*	0.14
Electrical conductivity (µS/cm)	152	10–623	194 (139)	152 (92)*	231 (80)	101 (58)*	0.15*					−0.08	0.43*
Dissolved oxygen (%)	85	25–138	92 (18)	73 (16)*	72 (17)	73 (16)	0.06						−0.05
Redox potential (mV)	86	−39–275	118 (79)	85 (28)	75 (11)	92 (34)	0.00						

Abbreviations: m.a.s.l. = meters above mean sea level, NTU = nephelometric turbidity units, mean = arithmetic mean, SD = standard deviation, Corr. ratio = correlation ratio, *B*. *p*. = *B*. *pseudomallei*, Tur = turbidity, Temp = temperature, pH = acidity, EC = electrical conductivity (proxy for salinity), DO = dissolved oxygen, ORP = redox potential. N = 67; exceptions: turbidity (n = 66), median, minimum and maximum of altitude in the rainy season (n = 31). The Bravais-Pearson correlation coefficient (r) is given for directional correlations between physico-chemical parameters, the correlation ratio (*η*^2^) for undirectional correlations between the presence of *B*. *pseudomallei* and physico-chemical parameters, range from 0 (no correlation) to 1 (perfect correlation). Statistical tests: seasonal comparison: paired t-test (n = 31 pairs, for turbidity n = 30 pairs), regional comparison: t-test, correlation ratio: t-test, Bravais-Pearson correlations: Pearson test; *statistically significant correlations or differences between groups, p < 0.01.

## Discussion

We detected *B*. *pseudomallei* in more than half (57%) of the investigated rivers, which indicates a widespread distribution of the pathogen in Laos. To characterise rivers as potential reservoirs or carriers for *B*. *pseudomallei*, we analysed the seasonal dynamics of its occurrence in both river water and superficial near-riparian sediments. If rivers were reservoirs, i.e. permanent habitats for *B*. *pseudomallei*, we would expect to find the pathogen primarily and perennially in the uppermost streambed sediments which harbor the majority of bacterial biomass in rivers^14^, and resuspended in the water column under conditions of increased turbulence, e.g. during floods. However, in accordance with the highest seasonal incidence of melioidosis^15^, we detected *B*. *pseudomallei* predominantly in the rainy season while *B*. *pseudomallei*-positive sediments were rare and usually linked to *B*. *pseudomallei*-positive water samples. These findings suggest that rivers are potential carriers for *B*. *pseudomallei*, and streambed sediments do not seem to be permanent habitats for this bacterium although the occurrence of *B*. *pseudomallei* in deeper midstream sediments is unknown. Nevertheless, the role of rivers and other freshwater bodies^16^ in the seasonal transmission of melioidosis might be underestimated, despite the fact that melioidosis cases have rarely been associated with exposure to river water^17^.

The most likely source of *B*. *pseudomallei* in rivers are its known reservoir, tropical soils^6^. Being present down to at least 90 cm depth^18^, the pathogen is likely to be mobilised with eroded soil particles in surface and subsurface runoff and ultimately channeled into rivers. As suggested by *B*. *pseudomallei*-positive filters of different pore sizes, the pathogen may be transported free-floating or attached to suspended particles of various sizes. Under conditions of high discharge, *B*. *pseudomallei* may be washed onto the soil of flood plains or infiltrate alluvial banks and aquifers downriver^19^ and be washed away again, especially during periods of heavy rainfall. In the Mekong basin, 90% of the annual precipitation (~1000 to 2800 mm) occurs during the southwest monsoon^20,21^, when *B*. *pseudomallei* was most common. Rain and, consequently, runoff are the main erosional forces of climatic origin in humid tropical regions, and intensive rainfall has been associated with increased erosion and suspended sediment load in the Mekong area^22,23^. Accordingly, we detected *B*. *pseudomallei* predominantly in particle-rich water, as observed in previous studies^10,12,13^.

However, *B*. *pseudomallei* was absent in the turbid rivers of the Northern Highlands, where sloping lands are particularly susceptible to erosion due to extensive land-use changes^23,24^. We can only speculate about the reasons why we detected the pathogen exclusively in the Mekong plain, although samples from melioidosis patients have been referred to the Mahosot Hospital Microbiology Laboratories from almost all Lao provinces (unpublished observations). Methodological considerations include the definition of the north-south boundary, which was based on limited sources, but classifying the southern-most northern stations as southern stations did not change the statistical significance of the north-south contrast regarding the presence of *B*. *pseudomallei*. Bias caused by non-random sampling (for reasons of accessibility) and bacterial loads below the detection limits of our methods cannot entirely be excluded. However, we applied two independent detection methods including post-enrichment PCR, which previously proved to be the most sensitive method for the detection of *B*. *pseudomallei* in environmental samples^25^. The absence or low numbers of *B*. *pseudomallei* may be a consequence of contrasting climate, geological substrates, soil types, and land-use in the Northern Highlands compared to the Mekong plains in southern Laos. The higher proportion of irrigated rice cultivation (paddy rice) and industrial agricultural plantations in the Mekong plain in contrast to slash-and-burn cultivation in the north^24^, for instance, as well as regionally distinctive parameters such as lower temperature or higher salinity values of northern river water (own data and^26^), might be aspects of a non-permissive environment for *B*. *pseudomallei*. However, direct conclusions cannot be drawn based on single water samples from rivers with large catchment areas, as *B*. *pseudomallei* might originate from various sources upriver, having been associated with a broad range of soil types and land-covers^12,27–30^. For this reason, analyses of relationships between *B*. *pseudomallei* in rivers and environmental factors in a catchment area are considered to be most conclusive at the sub-catchment or meso-scale (10–100 km^2^)^12,31^, and remain to be investigated in Laos and elsewhere.

We provide evidence that rivers are potential carriers for *B*. *pseudomallei*, as has been shown for other soil organisms^32^, but likely not permanent reservoirs for this pathogen. Rivers facilitate the dispersal of *B*. *pseudomallei* in the environment, possibly over long distances and to previously non-endemic areas. Thus, rivers are potential sentinels to explore the presence of *B*. *pseudomallei* in catchment areas, particularly during periods of intensive erosion and high discharge. Moreover, rivers may be useful to track potential sources and monitor the spatiotemporal dynamics of aquatic dispersal of *B*. *pseudomallei* and other environmental pathogens in a watershed and beyond.

## Methods

### Sample collection and processing

We investigated 36 stations at 23 perennial rivers, including the Mekong, in Laos between 15°N and 20°N in the dry (March) and rainy (July) seasons in 2016. The choice of rivers and sites was based on a broad geographical coverage of Laos and a range of differently sized direct or indirect tributaries to the Mekong. Several rivers were sampled at multiple sites along their course (Table 1). We collected unreplicated surface water samples from the riverside (near-riparian zone) using 1.5 L PET drinking water bottles (triple-rinsed with water from the sampling site), and from a mixed composite sample across the river at two southern Mekong stations. Wherever feasible, we collected bulk samples from the top 10 cm of near-riparian streambed sediment using a 102 cm^3^ hand-held steel cylinder, and kept them in sterile, ziplocked plastic bags. On-site physico-chemical measurements included altitude and geographical coordinates using a GPS device (Garmin Oregon 650t), water turbidity using a nephelometric turbidity meter (Eutech TN100), and water temperature, acidity (pH), electrical conductivity (a proxy for salinity), dissolved oxygen, and redox potential using a portable multi-probe (YSI-556). All samples were transported in a cool box with ice packs. One to four days post-sampling, we manually homogenised the sediment samples and conducted vacuum filtration at the Mahosot Hospital Microbiology Laboratories with 500 mL (dry season) and 250 mL (rainy season) of water, using an electrical pump, 1-L glass flasks, a stainless-steel funnel (Whatman) and two membrane filters applied in succession: a pre-filter (5.0 µm pore size) and a main filter (0.2 µm pore size) (cellulose acetate, 47 mm diameter, Sartorius). The equipment was cleaned with 70% ethanol and sterile water between samples.

### Microbiological methods

To detect *B*. *pseudomallei* on water filters and in sediment, we applied two independent methods: conventional culture techniques and PCR after an enrichment step, a sensitive approach for the detection of *B*. *pseudomallei* in low-abundance environments^25^. All microbiological analyses were conducted at the Mahosot Hospital Microbiology Laboratories in Class II Biosafety Cabinets.

### Culture

Water filters (one pre-filter and one main filter per sampling site) were placed surface-up on Ashdown’s agar while sediment samples were prepared as described previously for soil^33^. In short, 100 g of homogenised sediment were mixed with 100 mL of sterile water in sterile, ziplocked plastic bags and left to settle at room temperature overnight before different volumes of supernatant (10, 100 and 500 µL) were spread on Ashdown’s agar. In addition, 1 mL supernatant was enriched with 9 mL of selective TBSS-C50^34^ at 40 °C for 48 h, and 10 µL of the enriched fluid incubated on Ashdown’s agar. All samples were incubated at 40 °C in air for up to 4 days with daily inspection (median 3 days, range 2–4 days). Suspect colonies were tested by agglutination with a latex reagent specific for the 200-kDa exopolysaccharide of *B*. *pseudomallei*^35^ resistance to colistimethate and susceptibility to amoxicillin-clavulanic acid, and latex-positive isolates with these characteristics were confirmed by API 20NE (BioMérieux, Basingstoke, UK)^36^ and a specific PCR based on^37^ with the following modifications: 20 µL reaction mixture containing final concentrations of 0.5 µM primers LPW13372 and LPW13373, 2 mM MgCl_2_, 200 µM each dNTP, 1 U Platinum Taq (Invitrogen) and 1x Platinum PCR buffer. Thermocycler conditions were 95 °C for 10 minutes, followed by 40 cycles of 95 °C for 30 seconds, 60 °C for 45 seconds and 72 °C for 60 seconds, and a final extension of 72 °C for 10 minutes.

### Pre-enrichment and DNA extraction

Pre-enrichment and DNA extraction were conducted as described previously^25^ with some modifications: Entire pre- and main filters and 20 g of homogenised sediment were immersed separately in 20 mL of modified Ashdown’s broth, and, after shaking the sediment samples at 12 × g for 2 h, vortexed and incubated at 37 °C in air for 42 h. The enriched samples were kept at −20 °C, defrosted and vortexed shortly before DNA extraction. After settling for 20 min, the liquid phase of the enriched sediments was centrifuged at 700 × g for 2 min and mixed with 150 µL of 3.5 mg/L aurintricarboxylic acid. Then, all enriched samples were centrifuged at 3220 × g for 45 min and DNA extracted from the sedimentation using the MoBio PowerSoil DNA isolation kit according to the manufacturer’s instructions with an additional cell lysis step (incubation with proteinase K at 55 °C for 30 min)^25^.

### PCR

We applied a specific real-time PCR assay targeting a 115-base-pair region in the open-reading-frame 2 of the type III secretion system gene cluster (TTS1) of *B*. *pseudomallei* as described in^25^ with 500 nM primers BpTT4176F and BpTT4290R, 250 nM probe BpTT4208P (Biosearch Technologies) and 1 U Platinum Taq (Invitrogen), using a Rotor-Gene 6000 system (Qiagen) with 45 amplification cycles. Two positive controls (10^3^ and 10^4^ genome equivalents) and negative controls were included in every PCR run and showed the expected results. To control for PCR inhibition, 10^5^ copies of *Orientia tsutsugamushi* 47-kDa plasmid was amplified with *O*. *tsutsugamushi* specific primers and probe^25^. Inhibition was assumed to be absent if the spiked DNA amplified within ±2 Ct values from the positive inhibition controls which was the case for all samples (occasionally after dilution).

### Mapping and statistics

Maps were created with ArcGIS 10.3 and Adobe Illustrator CS6 using GPS coordinates of the sampling sites, elevation data (U.S. Geological Survey, https://earthexplorer.usgs.gov; Central Intelligence Agency, https://www.cia.gov/library/publications/the-world-factbook/index.html) and rivers/lakes/country shapefiles provided by the Centre for Development and Environment (CDE), CDE Lao Country Office. The geographical categories north (Northern Highlands) and south (Mekong plain and Annamite mountains, corresponding to the political centre and south) were based on a physio-geographical classification^38^, a geological map^39^ and topographic features. Statistical analyses were computed with Stata 14 and R 3.4.

### Data availability

The datasets generated and analysed during the current study are available from the corresponding author on reasonable request.
